# Validity of Platelia Aspergillus IgG and Aspergillus Precipitin Test To Distinguish Pulmonary Aspergillosis from Colonization

**DOI:** 10.1128/spectrum.03435-22

**Published:** 2022-12-08

**Authors:** Kyota Shinfuku, Junko Suzuki, Keita Takeda, Masahiro Kawashima, Yoshiteru Morio, Yuka Sasaki, Hideaki Nagai, Akira Watanabe, Hirotoshi Matsui, Katsuhiko Kamei

**Affiliations:** a Center for Pulmonary Diseases, National Hospital Organization Tokyo National Hospital, Tokyo, Japan; b Division of Clinical Research, Medical Mycology Research Center, Chiba University, Chiba, Japan; Universidade de Sao Paulo

**Keywords:** chronic pulmonary aspergillosis, allergic bronchopulmonary aspergillosis, *Aspergillus* colonization, Platelia *Aspergillus* IgG, *Aspergillus* precipitin test

## Abstract

When Aspergillus, an ubiquitous, saprophytic fungus, is detected in respiratory tract specimens collected from chronic respiratory disease patients, it is important to determine whether it is a true infection or colonization. We investigated the usefulness of the Bio-Rad Platelia Aspergillus IgG (Platelia Aspergillus IgG) enzyme-linked immunosorbent assay (ELISA) method and the Aspergillus precipitin test to distinguish pulmonary aspergillosis from colonization. Between January 2017 and November 2021, 51 confirmed, untreated pulmonary aspergillosis (33 chronic pulmonary aspergillosis [CPA] and 18 allergic bronchopulmonary aspergillosis [ABPA]) and 77 colonization patients were included in this study. At first, the conventional cutoff value was utilized in assessing the validity of the two antibody tests for distinguishing pulmonary aspergillosis from colonization. The Platelia Aspergillus IgG cutoff value was then reevaluated to fit this situation. Finally, differences in test accuracy dependent on Aspergillus species were assessed for both antibody tests by comparing cases with Aspergillus fumigatus complex and those with non-*fumigatus* Aspergillus complex. Both antibody tests demonstrated significantly higher positive rates for pulmonary aspergillosis (*P* < 0.0001) than colonization. The cutoff value should be 15.7 arbitrary units (AU)/mL to best distinguish infection from colonization, which was higher than the conventional value of 10 AU/mL. The diagnostic sensitivity of Platelia Aspergillus IgG for the non-*fumigatus* Aspergillus complex was inferior to the A. fumigatus complex (*P = *0.019). In conclusion, both Aspergillus antibody tests were valid to distinguish infection from colonization, although we should note the higher cutoff value for Platelia Aspergillus IgG and the lower sensitivity in cases of non-*fumigatus* Aspergillus infection.

**IMPORTANCE** Pulmonary aspergillosis is the most common pulmonary fungal infection. However, Aspergillus is a ubiquitous, saprophytic fungus; it can be detected in respiratory specimens even in the absence of infection. Especially since Aspergillus is detected in respiratory specimens collected from patients with chronic respiratory disease, it is important to determine whether it is true infection or colonization. We investigated the validity of the Platelia Aspergillus IgG ELISA method and the Aspergillus precipitin test to distinguish pulmonary aspergillosis from colonization. Both antibody tests were considered useful in differentiating true infection from colonization in respiratory practice. The appropriate cutoff value for Platelia Aspergillus IgG was higher than the conventional value, and it was also noted that the sensitivity of both antibody tests for non-*fumigatus* Aspergillus complex was low. This study will be significant in real-world clinical practice of pulmonary aspergillosis using antibody tests in respiratory care.

## INTRODUCTION

Pulmonary aspergillosis is the most common pulmonary fungal infection globally and primarily occurs in patients with underlying pulmonary diseases who are exposed to airborne Aspergillus spores. The immune status of the host and the toxicity and quantity of the fungi affect disease onset (1). Chronic pulmonary aspergillosis (CPA) and allergic bronchopulmonary aspergillosis (ABPA) are two of the most common clinical manifestations of pulmonary aspergillosis. The prevalence of CPA and ABPA is estimated to be over 3 and 4.8 million, respectively (2, 3).

Since Aspergillus species are ubiquitous, saprophytic fungi in the environment, anyone may inhale fungal spores (4, 5). Then, pulmonary aspergillosis occurs in patients with chronic respiratory disease. When Aspergillus is detected in patients with chronic respiratory disease, it is important to distinguish true infection from colonization. Aspergillus IgG antibody tests may play a role in diagnosing pulmonary aspergillosis (6, 7). However, it has been reported that patients with cystic fibrosis (CF) are exposed to Aspergillus in the environment and have higher IgG levels than healthy individuals (8), and it is possible that patients with chronic respiratory disease may similarly produce antibodies due to exposure and have higher levels than normal individuals. When Aspergillus is detected in respiratory tract specimens from patients with chronic respiratory disease, whether antibody tests can discriminate colonization from pulmonary aspergillosis is a very important question for which few reports have been reported (9, 10).

The Aspergillus precipitin test and enzyme-linked immunosorbent assays (ELISAs) are two major methods to detect Aspergillus IgG antibodies. Currently, commercial Aspergillus IgG ELISAs are produced by Immulite (Germany) (11), Bio-Rad (France) (12), ImmunoCAP (Sweden) (13), and Bordier (Switzerland) (14). Bio-Rad Platelia Aspergillus IgG (Platelia Aspergillus IgG) has been directly compared with the Aspergillus precipitin test and was proven more sensitive (15). However, this study included cases after initiation of antifungal drugs, which would have affected antibody titers, and a control group was not set.

Diagnosis of untreated pulmonary aspergillosis and discrimination of colonization in cases of chronic respiratory diseases are important tasks in respiratory clinical practice. In this study, we investigated the validity of antibody tests for the diagnosis of pulmonary aspergillosis and the discrimination of colonization using the Aspergillus precipitin test and Platelia Aspergillus IgG.

In addition, we evaluated the diagnostic accuracy of Platelia Aspergillus IgG for non-*fumigatus* Aspergillus complex infection in this study. Both antibody tests were designed to detect Aspergillus fumigatus complex and are expected to be less sensitive for non-*fumigatus* Aspergillus complex (16, 17), which caused 15 to 40% of pulmonary aspergillosis (18–20). We previously demonstrated significantly lower sensitivity of the Aspergillus precipitin test for CPA with non-*fumigatus* Aspergillus complex (19).

## RESULTS

### Patient characteristics.

We examined the antibody titers of 51 pulmonary aspergilloses, including 33 CPA and 18 ABPA patients, and 77 colonization patients (Table 1). In the pulmonary aspergillosis group, the mean age was significantly lower as a result of the lower mean age of the ABPA patients, and the proportion of males was significantly higher. Four cases of pulmonary aspergillosis were diagnosed by histological samples. A. fumigatus complex cases accounted for approximately 70% of the Aspergillus species in each group.

**TABLE 1:** Characteristics of patients (*n* = 128)

Characteristic	Data for all patients (*n* = 128)	Data for patients with:		*P* value
		Pulmonary aspergillosis (*n* = 51)^*f*^	Colonization (*n* = 77)	
Age (yrs)^*a*^	68.9 ± 12.4	64.8 ± 15.4 (CPA, 68.7 ± 13.2; ABPA, 57.6 ± 17.0)	71.7 ± 9.1	0.027
Male/female^*b*^	68 (53.1)/60 (46.9)	33 (64.7)/18 (35.3)	35 (45.5)/42 (54.5)	0.032
Underlying pulmonary diseases^*b*^^,^^*d*^	128 (100)	51 (100)	77 (100)	>0.999
Nontuberculous pulmonary infection	46 (36.0)	9 (17.6)	37 (48.1)	
Bronchiectasis	26 (20.3)	13 (25.5)	13 (16.9)	
Prior pulmonary tuberculosis	22 (17.2)	13 (25.5)	9 (11.7)	
Chronic obstructive pulmonary disease	19 (14.8)	9 (17.6)	10 (13.0)	
Bronchial asthma	18 (14.1)	16 (31.4)	2 (2.6)	
Interstitial lung disease	6 (4.7)	1 (2.0)	5 (6.5)	
Lung cancer	1 (0.8)	0 (0)	1 (1.3)	
Drug use of immunosuppression^*b*^	14 (10.9)	4 (7.8)	10 (13.0)	0.404
Corticosteroids/immunosupressants	10 (7.8)/4 (3.1)	2 (3.9)/2 (3.9)	8 (10.4)/2 (2.6)	
Specimen type^*b*^				
Sputum	100 (78.1)	37 (72.5)	63 (81.8)	
Bronchoscope samples	25 (19.5)	11 (21.6)	14 (18.2)	
Tissue sample	3 (2.3)	3 (5.9)	0 (0)	
Type of samples^*b*^				
Culture-positive samples	124 (96.9)	47 (92.2)	77 (100)	
Histological samples	4 (3.1)	4 (7.8)	0 (0)	
Time from specimen collection to antibody test (days) ^*c*^^,^^*e*^	0 (−8 to 0)	−4 (−8 to 0)	0 (−5 to 0)	
Aspergillus species^*b*^				
A. fumigatus complex	92 (71.9)	36 (70.6)	56 (72.7)	0.717
A. niger complex	28 (21.9)	9 (17.6)	19 (24.7)	
A. terreus complex	2 (1.6)	1 (2.0)	1 (1.3)	
A. flavus complex	1 (0.8)	1 (2.0)	0 (0)	

a Data are presented as mean ± SD.

b Data are presented as no. (%).

c Data are presented as median (interquartile range).

d Includes duplicate cases.

e Respiratory tract specimens included sputum, bronchoscopy, and tissue samples.

f For CPA, *n* = 33; for ABPA, *n* = 18. CPA, chronic pulmonary aspergillosis; ABPA, allergic bronchopulmonary aspergillosis.

### Comparison of antibody-positivity rates between pulmonary aspergillosis and colonization.

Both antibody tests indicated a significantly higher positive rate in pulmonary aspergillosis (*P < *0.0001) (Table 2) than in colonization. Platelia Aspergillus IgG had sensitivity of 76.5% and specificity 89.6%, and the Aspergillus precipitin test had sensitivity of 62.7% and specificity of 88.3%. There was no significant difference in the diagnostic accuracy between the two tests. The sensitivity of Platelia Aspergillus IgG (76.5%) seems slightly higher than that of the Aspergillus precipitin test (62.7%).

**TABLE 2:** Diagnostic accuracy of antibody tests to distinguish pulmonary aspergillosis from colonization

Diagnosis	Platelia Aspergillus IgG result		*P* value	Aspergillus precipitin test result		*P* value
	No. (%) positive	No. (%) negative		No. (%) positive	No. (%) negative	
Pulmonary aspergillosis (*n* = 51)	39 (76.5)	12 (23.5)		32 (62.7)	19 (37.3)	0.131
CPA^*b*^ (*n* = 33)	25 (75.8)	8 (24.2)		22 (66.7)	11 (33.3)	0.414
ABPA^*b*^ (*n* = 18)	14 (77.8)	4 (22.2)		10 (55.6)	8 (44.4)	0.289
Colonization (*n* = 77)	8 (10.4)	69 (89.6)		9 (11.7)	68 (88.3)	0.797
Test accuracy of diagnosis and discrimination^*a*^			<0.0001			<0.0001

a Chi-square test was performed from positive rate of pulmonary aspergillosis and negative rate of colonization.

b CPA, chronic pulmonary aspergillosis; ABPA, allergic bronchopulmonary aspergillosis.

### Platelia Aspergillus IgG cutoff values and differences in antibody titers by clinical forms.

Table 3 shows the cutoff values and area under the curve (AUC) based on the receiver operating characteristic (ROC) curve for Platelia Aspergillus IgG when patients with colonization are used as a control. The AUC for all cases was 0.872, and the cutoff value by Youden index was 15.7 arbitrary units (AU)/mL, with a sensitivity of 74.5% and specificity of 94.8%. In addition to all cases, the study was conducted with CPA and ABPA. The AUC of CPA was 0.886, and the cutoff value was 16.4 AU/mL. The AUC of ABPA was 0.847, and the cutoff value was 13.8 AU/mL. The cutoff value should be set higher to distinguish CPA from colonization compared with ABPA.

**TABLE 3:** AUC and cutoff value based on ROC curve for Platelia Aspergillus IgG^*a*^

Patient diagnosis	AUC (95% CI)	*P* value	Cutoff value (AU/mL)	Sensitivity (%)	Specificity (%)
All patients (*n* = 128)	0.872 (0.804–0.941)	<0.0001	15.7	74.5	94.8
CPA/colonization (*n* = 110)	0.886 (0.813–0.960)	<0.0001	16.4	75.8	94.8
ABPA/colonization (*n* = 95)	0.847 (0.717–0.976)	<0.0001	13.8	77.8	90.9

a AUC, area under the curve; ROC, receiver operating characteristic; CPA, chronic pulmonary aspergillosis; ABPA, allergic bronchopulmonary aspergillosis.

**FIG 1:**
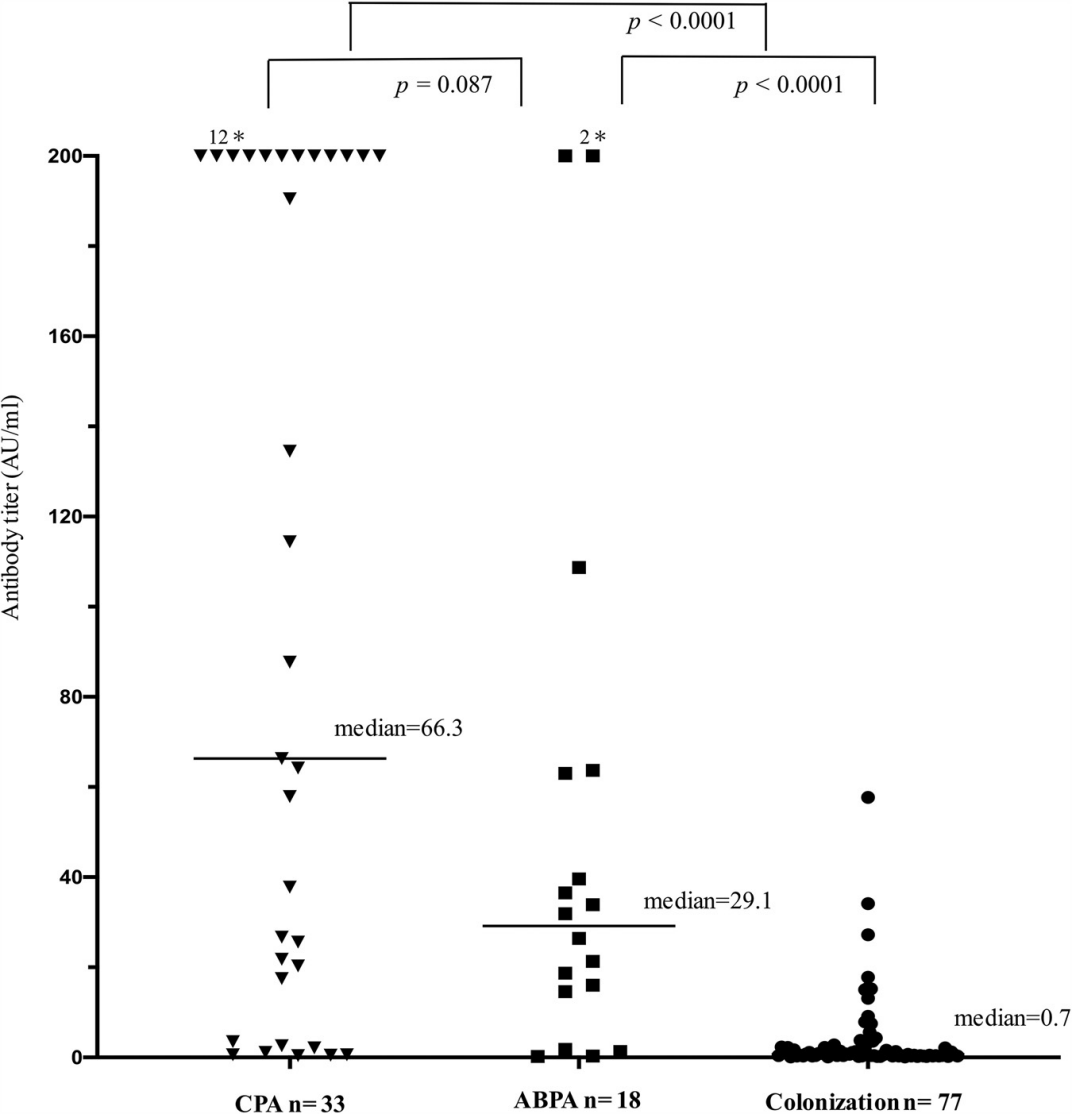
Comparison of antibody titers of chronic pulmonary aspergillosis (CPA), allergic bronchopulmonary aspergillosis (ABPA), and colonization. No significant difference was found, but the median antibody titer of CPA was higher than that of ABPA (*P = *0.087). The colonization group had significantly lower antibody titers than the other groups (*P < *0.0001). *, number of values >200 AU/mL. CPA, chronic pulmonary aspergillosis; ABPA, allergic bronchopulmonary aspergillosis.

Figure 1 shows the distribution and median antibody titers, compared by clinical forms. Although no significant difference was found, the median antibody titer of 66.3 AU/mL for CPA showed a higher trend than 29.1 AU/mL for ABPA (*P = *0.087). Antibody titers of the colonization group were found to be lower than those of the other two groups (*P < *0.0001).

### Comparison of test accuracy dependent on species.

Table 4 shows the difference in diagnostic accuracy between the A. fumigatus complex (*n* = 92) and non-*fumigatus* Aspergillus complex (*n* = 31). The Aspergillus niger complex (*n* = 28) was the most common non-*fumigatus* Aspergillus (90.3%). In Platelia Aspergillus IgG, the diagnostic sensitivity of pulmonary aspergillosis (83.3%) was significantly higher (*P = *0.019) for patients with A. fumigatus complex isolation than those with non-*fumigatus* Aspergillus complex isolation (45.5%).

**TABLE 4:** Comparison of diagnostic accuracy between Aspergillus species

Test and diagnosis	A. fumigatus complex (*n* = 92)^*a*^	Non-*fumigatus* Aspergillus complex (*n* = 31)^*a*^	*P* value
Platelia Aspergillus IgG			
Pulmonary aspergillosis	30/36 (83.3)	5/11^*b*^ (45.5)	0.019
CPA^*d*^	18/21 (85.7)	5/10 (50)	
ABPA^*d*^	12/15 (80)	0/1 (0)	
Colonization	6/56 (10.7)	2/20^*b*^ (10)	>0.999
Aspergillus precipitin test			
Pulmonary aspergillosis	24/36 (66.7)	4/11^*c*^ (36.4)	0.091
CPA^*d*^	16/21 (76.2)	4/10 (40)	
ABPA^*d*^	8/15 (53.3)	0/1 (0)	
Colonization	6/56 (10.7)	3/20^*c*^ (15)	0.690

a Data are presented as no. of positive tests/total no. of tests (%).

b A. niger complex, *n* = 9; A. terreus complex, *n* = 1; A. flavus complex, *n* = 1.

c A. niger complex, *n* = 19; A. terreus complex, *n* = 1.

d CPA, chronic pulmonary aspergillosis; ABPA, allergic bronchopulmonary aspergillosis.

## DISCUSSION

In this study, colonization patients with underlying chronic respiratory diseases in whom Aspergillus was detected in respiratory tract specimens were used as a control group, and we investigated the usefulness of antibody tests for the diagnosis of pulmonary aspergillosis. Both Platelia Aspergillus IgG and Aspergillus precipitin tests were valid in distinguishing Aspergillus infection from colonization.

Colonization was defined as a case in which Aspergillus was detected in the respiratory tract specimen from a patient with chronic respiratory disease, no symptom persistence, and image change suspicious for pulmonary aspergillosis for 6 months thereafter, and it was included in this study when an antibody test was submitted within 3 months of respiratory tract specimen detection. Following this definition, specificity was maintained at 89.6% for Platelia Aspergillus IgG and 88.3% for the Aspergillus precipitin test. However, colonization, in general, is not clearly defined and varies from report to report (4). Guitard et al. defined colonization as two cultures positive within 6 months without information on chronic respiratory disease, and they reported a specificity of 54.3 to 60% for Platelia Aspergillus IgG (9). On the other hand, Uffredi et al. defined colonization as one positive culture from a respiratory tract specimen with or without chronic respiratory disease, and they reported a specificity of 100% for the Aspergillus precipitin test (10). The specificity of the antibody test is expected to vary depending on the presence or absence of underlying chronic respiratory disease and the number and timing of respiratory tract specimen detections.

The sensitivity of Platelia Aspergillus IgG was 76.5% overall and 83.3% in A. fumigatus complex only, and that of the Aspergillus precipitin test was 62.7% overall and 66.7% in the A. fumigatus complex. There was no significant difference, but Platelia Aspergillus IgG was more sensitive, consistent with previous reports (15). The first reason for the higher sensitivity with Platelia Aspergillus IgG is the difference in test method. The Aspergillus precipitin test, which subjectively determines the presence or absence of a sedimentation line, is considered to have limitations in both sensitivity and specificity (21). Second, there are differences in the antigens used in the two tests. The Aspergillus precipitin test uses purified antigens from the culture extract, while Platelia Aspergillus IgG uses recombinant antigens that are highly sensitive synthesized proteins (22).

Next, we determined the cutoff values of the antibody titers for each clinical form, with cases of colonization as controls. It has been noted that using healthy subjects as controls to establish cutoff values for the ELISA method may differ from optimal cutoff values in the respiratory practice setting (15, 23). Page et al. reported cutoff values of Platelia Aspergillus IgG as low as 1.5 AU/mL when healthy subjects were used as controls (12). Bio-Rad used donor specimens provided by a French university hospital as controls and set the cutoff at 10 AU/mL. In this study, the overall cutoff value should be higher at 15.7 AU/mL due to the increase in false-positive cases in colonization, as shown in Fig. 1. In addition, there were cases of elevated Platelia Aspergillus IgG levels, even in patients who were considered to have colonization, suggesting that antifungal drug should not be easily initiated based on elevated antibody titers alone. The median antibody titer of CPA tended to be higher than that of ABPA, with a cutoff value of 16.4 AU/mL to distinguish CPA from colonization, higher than the cutoff value of 13.8 AU/mL of ABPA. While CPA is a fungal infection of the lungs with structural destruction, ABPA is a chronic airway disease caused by fungi confined to the mucus plugs in the airways, triggering an allergic reaction (6, 7). Therefore, ABPA is considered less invasive and has lower antibody titers and lower cutoff values than CPA.

Finally, we evaluated the diagnostic accuracy of antibody tests depending on the Aspergillus species, comparing the A. fumigatus complex with the non-*fumigatus* Aspergillus complex. We previously reported that the sensitivity of the Aspergillus precipitin test to CPA was 84.3% in the A. fumigatus complex and 37.9% in the non-*fumigatus* Aspergillus complex, significantly lower in the non-*fumigatus* Aspergillus complex (*P < *0.0001) (19). A Ugandan study of CPA complications in active pulmonary tuberculosis patients with persistent symptoms used Aspergillus*-*specific IgG/IgM immunochromatographic test (LD Bio) (24). In this study, 32 patients were included, the A. niger complex was more common (59.4%) than the A. fumigatus complex (21.9%), and the positive rate was low at 31.3%. No published data could be identified on the sensitivity of the ELISA method to the non-*fumigatus* Aspergillus complex. In this study, the sensitivity of Platelia Aspergillus IgG to the non-*fumigatus* Aspergillus complex was 45.5%, significantly lower than the sensitivity of 83.3% to the A. fumigatus complex (*P = *0.019). Antibody test antigens are designed to detect A. fumigatus, and the ELISA method is also expected to be less sensitive to non-*fumigatus* Aspergillus. This could explain the reasons for lower sensitivity of this study with Platelia Aspergillus IgG than in previous studies (9, 14, 15). The lower sensitivity of non-*fumigatus* Aspergillus complex suggests the importance of fungal culture of sputum and changes of symptoms and images to determine the overall necessity for treatment.

There were several limitations of this study. First, it was a single-center, retrospective study with a limited number of cases. Second, it is difficult to clearly distinguish colonization from contamination. Since the colonization cases were limited to those with underlying respiratory disease and antibody tests submitted within 3 months of respiratory tract specimen submission, it was difficult to include only cases with multiple positive respiratory tract specimens. We think that the possibility of contamination could be reduced by including cases in which respiratory tract specimens were detected more than once. Third, the coincident ratio between morphological and genetic identification at our institution was high at the complex level, 97.1% for A. fumigatus and 96.8% for A. niger (25), but identification of the species was done by morphological methods; genetic analysis could not be performed in this study.

In conclusion, antibody tests, both Platelia Aspergillus IgG and the Aspergillus precipitin test, are helpful in distinguishing pulmonary aspergillosis from colonization. When colonization patients with underlying chronic respiratory disease should be differentiated, the cutoff value of Platelia Aspergillus IgG should be higher at 15.7 AU/mL than the conventional value of 10 AU/mL. When CPA or ABPA are strongly suspected in cases of negative Aspergillus antibody, non-*fumigatus* Aspergillus complex would be checked by sputum culture for the pathogen.

## MATERIALS AND METHODS

### Study design and patients.

This retrospective diagnostic study was conducted at the National Hospital Organization (NHO) Tokyo National Hospital, Tokyo, Japan. We targeted only CPA, ABPA, and colonization in which Aspergillus culture-positive or pathological findings showed filamentous fungi with septa in respiratory tract specimens (sputum, bronchoscopy, or tissue samples) between January 2017 and November 2021. Respiratory tract specimens were cultured on Sabouraud dextrose agar (Kanto Kagaku, Tokyo, Japan) or potato dextrose agar (Kanto Kagaku, Tokyo, Japan) at 22 ± 2°C for ≤14 days after culturing at 35°C for 2 days. Filamentous fungi with septa were identified microscopically using Grocott’s or Fungiflora Y stain.

Species identification was only performed on culture-positive samples. Aspergillus species were identified morphologically by macroscopic and microscopic examination by one or two microbiologists (from a total of six microbiologists) with 10 to 20 years of experience in our laboratory. Once sufficient colony growth was observed 7 to 14 days after incubation, macroscopic examination was performed, and characteristics such as colony color, texture, exudates, pigmentation, and growth rate were recorded. Next, using the tape-mount method, hyphae, vesicles, phialide, and conidia were observed by microscopy and identified. Once the Aspergillus species was identified, a second microbiologist confirmed the identification of the fungal species. Since the identification of fungal species is limited to the *sensu lato* level by morphological techniques, the species were described at the complex level.

Then, to validate the antibody tests' accuracy of diagnosis in chronic respiratory diseases, Platelia Aspergillus IgG assays and Aspergillus precipitin tests were performed on the same sera within 1 month of Aspergillus detection in cases of pulmonary aspergillosis and within 3 months in cases of colonization. The time period between fungal detection and blood exam in colonization was allowed for 3 months because, due to absence of symptoms, there was often delay from order for the microbiological examination to collection of the sputum. In addition, antibody tests were often ordered after detection of Aspergillus in respiratory tract specimens. CPA, ABPA, or colonization was diagnosed according to the diagnostic criteria described below. We excluded patients who started antifungal drugs before serum samples were taken because antifungal drugs would have affected antibody titers (26, 27).

In this study, we first examined the diagnostic accuracy of pulmonary aspergillosis (CPA or ABPA) and colonization using Platelia Aspergillus IgG and the Aspergillus precipitin test, adopting the conventional cutoff value of 10 AU/mL. Then, we examined the appropriate cutoff values to best distinguish patients with CPA or ABPA from colonization as controls in the context of respiratory care. Finally, we also investigated the test accuracy of pulmonary aspergillosis, dependent on Aspergillus species.

### Clinical diagnosis.

CPA was diagnosed based on the Infectious Diseases Society of America and the European Respiratory Society guidelines (6, 7). In principle, CPA was diagnosed based on (i) chronic pulmonary and systemic symptoms, i.e., cough, sputum, hemoptysis, dyspnea, or fever; (ii) high-resolution computed tomography (HRCT) imaging findings of worsening cavitation, fugal ball, pericavitary infiltrates, pleural thickening, upper lobe fibrosis; and (iii) Aspergillus culture positive or filamentous fungi with septa were identified from respiratory tract specimens. All patients met the criteria for persistent symptoms and the typical HRCT image progression and culture or pathological findings and exclusion of alternative diagnosis.

ABPA was diagnosed based on the criteria reported by Agarwal et al. and Asano et al. (28, 29). In principle, ABPA was diagnosed based on (i) allergic predisposition and reaction to Aspergillus species, (ii) Aspergillus culture positive or filamentous fungi with septa that were identified from respiratory tract specimens, and (iii) existence of mucus plugs in central bronchi based on computed tomography or bronchoscopy. All patients met the criteria for allergic predisposition to Aspergillus and existence of mucus plugs and culture or pathological findings and exclusion of alternative diagnosis.

Colonization was defined as positive Aspergillus culture or filamentous fungi with septa detection from respiratory tract specimens of patients with underlying chronic respiratory diseases, such as bronchiectasis, nontuberculous pulmonary infection, prior pulmonary tuberculosis, chronic obstructive pulmonary disease (COPD), or interstitial lung disease, and without HRCT findings of new shadows or changes in existing structural destructive lesions suspicious for pulmonary aspergillosis. In addition, no persistent symptom or image changes were indicated for the next 6 months from fungal detection (4, 30).

To validate the accuracy of the antibody tests, microbiological finding as direct evidence of Aspergillus infection was necessary for the diagnosis of CPA, and serological finding was not adopted for the diagnostic criteria in this study. To distinguish between colonization and CPA, all patients were evaluated by HRCT and diagnosed separately by two respiratory physicians (K.S., J.S.) with more than 10 years of experience based on the above-described diagnostic criteria. The diagnoses of the cases requiring discussion were determined based on consensus.

### Aspergillus serological examination.

The Aspergillus precipitin test method is based on immunodiffusion analysis. We used an Aspergillus immunodiffusion system (Microgen Bioproducts Ltd., Camberley, UK) according to the manufacturer’s instructions (16). The Aspergillus precipitin test was considered positive when sedimentation lines were observed. On the other hand, as for ELISA, we used Platelia Aspergillus IgG (Bio-Rad Ltd., France) (17). The serum samples were diluted 400 times and distributed onto microplate wells sensitized with purified recombinant Aspergillus antigen. Furthermore, the secondary peroxidase-marked antibody was added to each microplate well. The optical density of the reaction was measured using a spectrophotometer set to 450/620 nm. The titer in units per milliliter (arbitrary units per milliliter) was calculated using the supplied calibration points (0 to 80 AU/mL). According to the manufacturer’s recommendation, we judged concentrations <10 AU/mL as negative and ≥10 AU/mL as positive. Samples with a titer of >80 AU/mL can be measured by dilution five times.

### Statistical analysis.

The Fisher test or chi-square test was used for comparisons between groups. The *t* test or Mann-Whitney U test was used to compare continuous variables. The cutoff value was determined using the Youden index of receiver operating characteristic (ROC) curve. The statistical analyses were performed using GraphPad Prism version 8.4.3 for Macintosh (GraphPad Software La Jolla, CA, USA), and the statistical significance was set at *P* values of <0.05.

### Ethics statement.

The protocol was approved by the institutional review board of the NHO Tokyo National Hospital, Tokyo, Japan (approval date, 9 February 2016; approval number 171129). Informed consent was obtained from the eligible patients.
